# Prevalence of burnout and associated factors among health professionals working in public health facilities of Dire Dawa city administration, Eastern Ethiopia

**DOI:** 10.3389/fpubh.2022.836654

**Published:** 2022-08-11

**Authors:** Fila Ahmed, Behailu Hawulte, Mohammed Yuya, Simon Birhanu, Lemessa Oljira

**Affiliations:** ^1^School of Public Health, College of Health and Medical Sciences, Haramaya University, Harar, Ethiopia; ^2^School of Nursing and Midwifery, Haramaya University, Harar, Ethiopia

**Keywords:** burnout, associated factors, health professional, Dire Dawa, Eastern Ethiopia

## Abstract

**Background:**

Burnout is a common condition among health workers, characterized by emotional tiredness, depersonalization, and a sense of low personal accomplishment. Ethiopia has major health workforce management challenges, including shortages, poor motivation, retention, and performance, and research evidence is limited for health professionals' burnout status, particularly in Eastern Ethiopia. Therefore, this study is aimed at determining the prevalence of burnout and associated factors among health professionals working at governmental health facilities in Eastern Ethiopia.

**Method:**

An institutional-based cross-sectional study was conducted among health professionals using structured self-administered validated questionnaires using the Maslach Burnout Inventory scale. Data were entered into Epi-Data version 3.1 and exported to SPSS version 22 for analysis. Multivariable logistic regression was used to determine the association between burnout and its predictors.

**Results:**

A total of 508 health professionals were approached, out of which 501 participated (a response rate of 98.4%). The magnitude of burnout was 54.1 with a 95% confidence interval of 49.9–58.0%. Working in a hospital (AOR = 3.55, 95%CI: 2.00, 6.33), age >/= 40 (AOR = 3.98, 95%CI:1.60, 9.89) and 30–39 years (AOR = 1.90, 95%CI:1.08, 3.34), being female(AOR = 2.41, 95%CI: 1.37, 4.25), being widowed (AOR = 3.39, 95%CI: 1.13, 10.18), having intention of leaving work (AOR = 2.28, 95%CI: 1.35, 3.87), using at least one substance (AOR = 2.24, 95%CI: 1.36, 3.69), having a 6–11 years of experience (AOR = 2.17, 95%CI: 1.15, 4.06), having no job supervision (AOR = 4.65, 95%CI: 2.07, 10.43), monthly payment <10,000 *Ethiopian Birr* (AOR = 5.69, 95%CI: 2.30, 14.07) and between 10,000 to 15,000 *Ethiopian Birr* (AOR = 2.74, 95%CI: 1.22, 6.15), working in Pediatric Unit (AOR = 3.28, 95%CI: 1.24, 8.70), and profession type (Midwifery, Public health officer, Medical Laboratory professionals) were factors significantly associated with burnout.

**Conclusion:**

Burnout affected more than half of the health professionals working in governmental health facilities in Dire Dawa. Health facility type, age, sex, marital status, intention to leave work, substance use, work experience, job supervision, monthly payment, profession type, and working unit were significantly associated predictors of burnout.

## Introduction

Burnout is a condition that is thought to be caused by unmanaged long-term working stress (1). Depersonalization, or sentiments of negativism or cynicism about one's employment; emotional exhaustion, or emotions of energy depletion or exhaustion; and decreased professional performance are the three dimensions of burnout (1). Burnout causes people to perform poorly at work, endangering both their patients and themselves (2).

Burnout is becoming more common among healthcare providers, who have been classified as a high-risk category (3). Employee happiness, mental and physical health, absenteeism rates, work productivity, and staff turnover are all negatively impacted by burnout syndrome. It can also have an impact on family duties and functions. Its consequences extend to threatening the health care system, including care quality, patient safety, and healthcare expenses (4, 5).

Because most studies on this topic are conducted in high-income nations, it is unclear how many people working in the health profession are burnt out globally (2). Furthermore, in low-and middle-income nations, the aggregate burden of burnout and its impact on healthcare providers remains unknown. Studies in different settings of the globe show that burnout in health professionals is precipitated by the health system, individual behavior, and health care seeker numbers (6–8).

In Sub-Saharan Africa, the health system suffers from a scarcity of healthcare professionals and overburdening of the available health professionals because of the increasing health-seeking behaviors in society, and physicians from Sub-Saharan Africa are migrating to the global workforce (9–11). Sub-Saharan African medical graduates made up around 6% of all overseas medical graduates entered the US workforce in 2015. Furthermore, more than 30% of physicians trained locally have gone to high-income nations in half of Sub-Saharan Africa's countries (9, 12).

In the last decade, Ethiopia has seen significant growth in primary health care services, including a large scale-up of health staff development and deployment. The number of health professionals in the population increased from 0.84 per 1,000 in 2010 to 1.5 per 1,000 in 2016. Ethiopia aims to meet the World Health Organization's (WHO) 2025 standard for Sub-Saharan Africa, which is a ratio of 2.3 health professionals per 1,000 people (13).

Plenty of interventions have been implemented to lower the occurrence of burnout among healthcare professionals around the globe (14, 15). The Ethiopian Federal Ministry of Health also introduced increments in incentives, rotation among frontline health professionals, and increased the number of health professional graduates to satisfy the growing demand of the health system, but the country has major health workforce management challenges, including shortages, poor motivation, retention, and performance (16).

Despite these increments in burnout among health professionals, only a few studies have been conducted in Ethiopia. Most of these studies were conducted in hospitals, and on single health professionals (6, 17). Further research into the factors of burnout among health professionals in various contexts is critical to halting the rising tide of burnout among health professionals. Therefore, the purpose of this study was to determine the magnitude of burnout and the factors that contribute to it among health professionals employed by the city of Dire Dawa.

## Methods and materials

### Study area, period, and design

A cross-sectional institutional study was undertaken in the Dire Dawa city administration's public health facilities from April 1 to April 30, 2020. Dire Dawa city is located in Ethiopia's eastern region, some 525 kilometers east of Addis Ababa, the country's capital. A total of 341,834 people live in the city administration. The city of Dire Dawa has 15 health centers, one general hospital, one referral hospital, and 768 health workers, according to the health office of the local administration (18).

### Study participants

The source population included all health professionals who worked in the Dire Dawa City administration public health institutions. The study population consisted of all health professionals who were available at work during the study period and were included in the sample. This study comprised health practitioners who hadworked for more than 6 months in Dire Dawa City administration public health institutions prior to data collection. During the data collection period, however, health professionals who were extremely ill or unable to reply were omitted from the study.

### Sample size determination

The study's minimum sample size was calculated by using both single population proportion formulas and a power approach based on results obtained from a similar study conducted in public hospitals in Amhara Regional State, Ethiopia (19). After that, by comparing the sample sizes obtained from those two formulas, the maximum sample size of 508 was chosen as the final sample size.

### Sampling procedure

In the city administration of Dire Dawa, there are two government hospitals and fifteen health centers. Each health facility's sample size was determined by proportionally allocating the calculated sample size depending on the number of health professionals on staff. The needed number of health professionals in each health facility was then picked using a simple random sample technique using the provided sampling frame (lists of health professionals with serial numbers) from each health institution's human resource department.

### Data collection tools and procedures

Self-administered questionnaires adapted from previously conducted studies were used to collect data (20–24). The questionnaire was prepared in English and has questions regarding socio-demographic status, personal factors, work-related factors, and burnout. The English version of Maslach's Burnout Inventory-Human Services Survey (MBI-HSS), adapted from previously conducted studies, was used to collect data on burnout among healthcare professional. The tool is comprised of 22 items regrouped into 3 subscales: emotional exhaustion (EE), depersonalization (DP), and personal accomplishment (PA). Each item was answered on a 7-point Likert scale (0: “never”, 1: “a few times a year or less”, 2: “once a month or less”, 3: “a few times a month”, 4: “once a week”, 5: “a few times a week”, and 6: “every day”). The MBI-HSS is a reliable and valid instrument to assess burnout 25, 30, 31 A previous study has indicated that even when used in another language (Spanish), the instrument produced high sensitivity and specificity, 92.2 and 92.1%, respectively (22–25). The data collection was conducted by thirteen trained diploma nurses and two supervisors recruited from health facilities that were not included in the study.

### Operational definition

The measure used to assess burnout among health workers includes 22 questions organized into three burnout domains: 5 questions about depersonalization, 8 questions about personal accomplishment, and 9 questions regarding emotional exhaustion. Each question has a response ranging from 0 = (“never”) to 6 = (“daily”) based on a 7-point Likert scale. Scores in each of the three subscales of burnout were divided into high, average, and low categories based on the cut-offs used in previous studies (22–25). Burnout refers to the value placed on the standard by the depersonalization, emotional exhaustion, and personal accomplishment subscales. So, if participants scored high in EE and DP but low in PA, they were considered burnt out.

Emotional exhaustion (EE) is defined as emotions of overextension as a consequence of one's job, with a score of 27 or more indicating high EE. Depersonalization (DP) is defined as emotional apathy and dehumanization of those who receive one's services, care, treatment, or instruction, with a score of 13 or more indicating high DP. Personal accomplishment (PA) is defined as sentiments of work stagnation, inefficiency, and underperformance, with a score of ≤ 31 indicating low PA (20–24).

### Data quality control

The principal investigator conducted 2 days of training for data collectors and supervisors about the data collection technique and tool. A pre-test was conducted on 5% of health workers in the *Bike primary hospital*, which is one of the non-selected health institutions in Dire Dawa city, and the necessary changes were implemented accordingly. The data were examined for completeness, accuracy, and consistency by supervisors.

### Data analysis procedures

The data were compiled, cleaned, and coded before being entered into Epi Data version 3.1 and exported to SPSS version 22 for analysis. The mean, median, and percentage were used to summarize the data. Tables and figures were used to present descriptive data. Using a bivariable logistic regression, crude odds ratios with 95% confidence intervals were calculated to analyze the relationship between each independent variable and the outcome variable. Variables having a *P*-value of <0.25 in the bivariable logistic regression were included in the multivariable logistic regression analysis. A Hosmer-Lemeshow goodness-to-fit model was tested for model fitness. Finally, adjusted odds ratios with 95% confidence intervals were calculated to assess the strength of the association, and variables with a *p*-value of <0.05 were considered statistically significant factors.

### Ethical approval and participant consent

The Institutional Health Research Ethical Review Committee (IHRERC) at Haramaya University gave ethical approval. The city administration of Dire Dawa approved the conduct of this study and sent a formal letter of support to the respective health facilities in the City. Informed, voluntary, written and signed consent was taken from each study participant after explaining the aim and potential benefit of the study and confidentiality. This research was carried out in line with the Helsinki Declaration.

## Results

### Socio-demographic characteristics of the study participants

A total of 501 healthcare workers took part in this study, with a response rate of 98.4%. Two hundred fifty-one (50.1%) of the respondents were male, and the respondents' average age was 32 years old, with a standard deviation of 6.4 years. One hundred eighty-four (36.7%) of the participants were Muslim in religion, and 222 (44.3%) of the participants were married. In terms of educational level, 291 (58.1%) of professionals were bachelor's degree holders and 238 (47.5%) were nurses in the profession. Two hundred four (40.7%) of the participants had 6–11 years of experience, and 271 (46.6%) of the participants had ≤ 10,000 *Ethiopian Birr* (ETB) monthly salary. Three hundred twenty-eight (65.5%) of the participants were urban residents (Table 1).

**Table 1:** The sociodemographic characteristics of healthcare professionals working at public health facilities in Dire Dawa City, Eastern Ethiopia, 2020 (n = 501).

**Variables**	**Categories**	**Number**	**Percentage**
**Age**	≤ 29 years	173	34.5
	30–39 years	267	53.3
	≥40 years	61	12.2
**Sex**	Male	251	50.1
	Female	250	49.9
**Religion**	Muslim	184	36.7
	Orthodox	172	34.3
	Protestant	145	28.9
**Marital status**	Married	222	44.3
	Single	200	39.9
	Divorced/Separated	54	10.8
	Widowed	25	5.0
**Educational status**	Diploma/Level 4	192	38.3
	Degree	291	58.1
	Masters	18	3.6
**Profession type**	Nurse	238	47.5
	Midwife	50	10.0
	Laboratory	55	11.0
	Pharmacy	53	10.6
	Public Health (HO)	38	7.6
	Others^*a^	67	13.4
**Work Experience**	≤ 5 years	123	24.6
	6–11 years	204	40.7
	>11 years	174	34.7
**Residence area**	Urban	328	65.5
	Rural	173	34.5

Others^*a^ = Specialist., medical doctor, IESO, anesthesia and Physiotherapy.

### Work-related factors

Four hundred forty-nine (89.6%) of participants had supervision in their current job, and 281 (56.1%) had ≤ 72 average working hours per week. Two hundred ninety-eight (59.5%) of the participants were not substance users. The majority of the participants, 290 (57.9%), worked at health centers, while 81 (16.2%) worked in the adult OPD. One hundred thirty-eight (27.5%) of the participants are currently working as—managers or have a position, and 166 (33.1%) had the intention of leaving their work (Table 2).

**Table 2:** Work-related factors of health professionals working at public health facilities in Dire Dawa City, Eastern Ethiopia, 2020 (n = 501).

**Variables**	**Categories**	**Frequency**	**Percent**
Having job supervision	Yes	449	89.6
	No	52	10.4
Average working hours per week	≤ 72 h	281	56.1
	≥72 h	220	43.9
Substance use	No	298	59.5
	Yes	203	40.5
Health facility type	Health center	290	57.9
	Hospital	211	42.1
Current working Unit or service delivery unit	Adult OPD	81	16.2
	Under 5 OPD	47	9.4
	Emergency care unit	55	11.0
	Medical unit	51	10.2
	Pediatric unit	36	7.2
	Surgical unit	22	4.4
	Gyn/Obs. unit	47	9.4
	Others^*b^	162	32.3
Monthly income (wage and incentive) (in)	<10,000 *Ethiopian Birr*	197	39.3
	10,000–15,000 ETB	229	45.7
	>15,000 *Ethiopian Birr*	75	15.0
Facility type	Health Center	290	57.9
	Hospital	211	42.1
Have Position or manager	Yes	138	27.5
	No	363	72.5
Intention of leaving the job	Yes	166	33.1
	No	335	66.9

Others^*b^ = MCH, ophthalmic, dental clinic, psychiatry unit, ART and TB clinic, and ICU.

### Prevalence of burnout among health professionals

**Figure 1:**
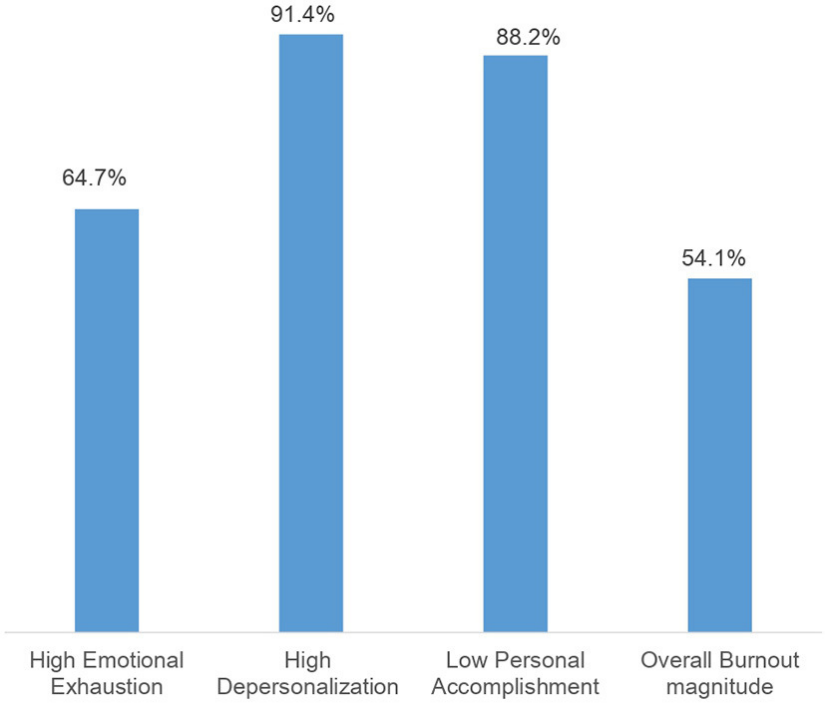
Magnitude of burnout among health professionals working at public health facilities in Dire Dawa city administration, Eastern Ethiopia 2020 (*n* = 501).

The prevalence of burnout was found to be 54.1% among health professionals working in Dire Dawa city administration public health facilities, with a 95% CI of 49.9–58.0%. Regarding components of burnout, 324 (64.7%) of the participants had high emotional exhaustion, 458 (91.4%) of the participants had high depersonalization, and 442 (88.2%) of the participants had low personal accomplishment (Figure 1).

### Factors associated with high burnout of health professionals

To determine the predictors of burnout among health professionals, bivariable and multivariable binary logistic regression models were used. In the bivariable analysis, average monthly income (payment), type of profession, intention of leaving a job, having job supervision, average working hours per week, facility type, sex, current marital status, residence area, substance use, total work experience, and current working unit had a significant association with health professional's burnout at *p*-value <0.05 whereas age, education, residence, working area, and having a job position were not significant at *p*-value <0.05 but included in the multivariable analysis, based on reviewing previous studies (6, 21, 25, 29, 32).

In multivariable analysis, health professionals working in hospitals had a 3.55 times greater chance of burnout than those working in health centers (AOR = 3.55, 95% CI: 2.00, 6.33). Participants over the age of 30 were more likely to have burnout, with >/= 40 years the most likely (AOR = 3.98, 95% CI: 1.60, 9.89) than ages 30–39 (AOR = 1.90, 95% CI: 1.08, 3.34). When compared to male health professionals, female health professionals had 2.41 times more chances of burnout (AOR = 2.41, 95% CI: 1.37, 4.25). Widowed health professionals had a 3.39 times greater risk of burnout (AOR = 3.39, 95% CI: 1.13, 10.18) compared to married health professionals.

Burnout was twice as common among health professionals who planned to leave their employment (AOR = 2.28, 95% CI: 1.35, 3.87) and those who used at least one substance (AOR = 2.24, 95% CI: 1.36, 3.69) as it was among their counterparts. Health professionals who had 6–11 years of work experience had a 2.17 times greater risk of burnout compared to health professionals who had 5 years of experience or less (AOR = 2.17, 95% CI: 1.15, 4.06). Health professionals who did not get job supervision had a 4.65 times higher risk of burnout than those who did (AOR = 4.65, 95% CI: 2.07, 10.43).

An average monthly payment of fewer than 10,000 ETB (AOR =5.69, 95%CI: 2.30, 14.07) and between 10,000 and 15,000 ETB (AOR =2.74, 95%CI: 1.22, 6.15) was a risk factor for burnout when compared to people who earn more than 15,000 ETB per month. In comparison to nurse professionals, midwives (AOR = 8.33, 95% CI: 3.11, 22.34), medical laboratories (AOR = 5.47, 95% CI: 2.06, 14.54), public health officers (AOR = 4.65, 95% CI: 1.85, 11.64), and other health professionals (AOR = 15.86, 95% CI: 6.00, 41.89) had higher burnout rates. Working in a pediatric unit increased the risk of burnout by 3.28 times compared to working in an adult OPD (AOR =3.28, 95% CI: 1.24, 8.70) (Table 3).

**Table 3:** Multivariable logistic regression of factors associated with burnout of healthcare professionals working at public health facilities of Dire Dawa City, Eastern Ethiopia, 2020 (*n* = 508).

**Variables**	**Categories**	**Burnout**		**COR (95% CI)**	**AOR (95% CI)**
		**Yes *N* (%)**	**No *N* (%)**		
Health facility type	Hospital	128 (60.7)	83 (39.3)	1.59 (1.11, 2.27)	3.55 (2.00, 6.33)***
	Health center	143 (49.3)	147 (50.7)	1	1
Age	≤ 29 years	86 (49.7)	87 (50.3)	1	1
	30–39 years	155 (58.1)	112 (41.9)	1.40 (0.95, 2.06)	1.90 (1.08, 3.34)*
	≥40 years	30 (49.2)	31 (50.8)	0.98 (0.55, 1.76)	3.98 (1.60, 9.89)**
Sex	Female	147 (58.8)	103 (41.2)	1.46 (1.03, 2.08)	2.41 (1.37, 4.25)***
	Male	124 (49.4)	127 (50.6)	1	1
Marital status	Married	114 (51.4)	108 (48.6)	1	1
	Single	105 (52.5)	95 (47.5)	1.05 (0.71, 1.54)	1.24 (0.71, 2.16)
	Divorced	33 (61.1)	21 (38.9)	1.49 (0.81, 2.73)	1.56 (0.75, 3.25)
	Widowed	19 (76.0)	6 (24.0)	3.00 (1.16, 7.80)	3.39 (1.13, 10.18)*
Educational level	Diploma	116 (60.4)	76 (39.6)	1.91 (0.72,5.05)	8.81 (1.99, 39.98)
	BSc degree	147 (50.5)	144 (49.5)	1.28 (0.49, 3.33)	7.46 (1.85, 30.10)
	MSc and above	8 (44.4)	10 (55.6)	1	1
Residence area	Rural	102 (59.0)	71 (41.0)	1.35 (0.93, 1.96)	1.63 (0.91, 2.90)
	Urban	169 (51.2)	159 (48.8)	1	1
Intention to leave work	Yes	105 (63.3)	61 (36.7)	1.75 (1.20,2.57)	2.28 (1.35, 3.87)**
	No	166 (49.6)	169 (50.4)	1	1
Substance use	Yes	122 (60.1)	81 (39.9)	1.51 (1.05, 2.16)	2.24 (1.36, 3.69)**
	No	149 (50.0)	149 (50.0)	1	1
Total work experience	<5 years	59 (48.0)	64 (52.0)	1	1
	6–11 years	124 (60.8)	80 (39.2)	1.68 (1.07, 2.64)	2.17 (1.15, 4.06)*
	>11 years	88 (50.6)	86 (49.4)	1.11 (0.70, 1.76)	1.38 (0.73, 2.62)
Having job supervision	No	39 (75.0)	19 (25.0)	2.81 (1.46,5.40)	4.65 (2.07,10.43)***
	Yes	232 (51.7)	219 (48.3)	1	1
Monthly income (wage and incentive)	≤ 10,000 ETB	121 (61.4)	76 (38.6)	5.69 (2.30,14.07)	5.69 (2.30,14.07)***
	1,000–15,000 ETB	116 (50.7)	113 (49.3)	2.74 (1.22, 6.15)	2.74 (1.22, 6.15)**
	>15,000 ETB	34 (45.3)	41 (54.7)	1	1
Weekly working hours	>72 h	135 (48.0)	146 (52.0)	1.75 (1.22, 2.51)	1.24 (0.69, 2.22)
	≤ 72 h	136 (61.8)	84 (38.2)	1	1
Profession	Nurse	105 (44.1)	135 (55.9)	1	1
	Midwife	32 (64.0)	18 (36)	2.25 (1.20, 4.24)	8.33 (3.11,22.34)***
	Laboratory	38 (69.1)	17 (30.9)	2.83 (1.51, 5.30)	5.47 (2.06, 14.54)**
	Pharmacy	28 (52.8)	25 (47.2)	1.42 (0.78, 2.58)	2.43 (0.93, 6.37)
	Public Health/HO	21 (55.3)	17 (44.7)	1.57 (0.79, 2.58)	4.65 (1.85, 11.64)**
	Others^*a^	47 (70.1)	20 (29.9)	2.98 (1.66, 5.33)	15.86 (6.00,41.89)***
Current working unit or Service delivery unit	Adult OPD	36 (44.4)	45 (55.6)	1	1
	Under 5 OPD	21 (44.7)	26 (55.3)	1.01 (0.49, 2.08)	1.16 (0.48, 2.78)
	Emergency Unit	35 (63.6)	20 (36.4)	2.19 (1.08, 4.42)	2.14 (0.93, 4.96)
	Medical Unit	23 (45.1)	28 (54.9)	1.03 (0.51, 2.08)	1.31 (0.53, 3.25)
	Pediatric Unit	23 (63.9)	13 (36.1)	2.21 (0.99, 4.97)	3.28 (1.24, 8.70)*
	Surgical Unit	8 (36.4)	14 (63.6)	0.71 (0.27, 1.89)	1.03 (0.33, 3.26)
	Gyn/Obs. Unit	25 (53.2)	22 (46.8)	1.42 (0.69, 2.92)	0.49 (0.16, 1.49)
	Others^*b^	100 (61.7)	62 (38.3)	2.02 (1.17, 3.46)	1.23 (0.51, 2.97)
Position (management)	No	205 (48.0)	158 (52.0)	1.42 (0.96, 2.10)	1.08 (0.63, 1.86)
	Yes	66 (61.8)	72 (38.2)	1	1
Duration of consultation time	>30 min	42 (55.3)	34 (44.7)	1	
	≥20 min	119 (77.3)	35 (22.7)	1.64 (1.06–2.55)	1.45 (0.87–2.42)
	<20 min	244 (67.4)	118 (32.6)	1	

Others^*a^ = Specialist., medical doctor, IESO, anesthesia and Physiotherapy; Others^*b^ = MCH, ophthalmic, dental clinic, psychiatry unit, ART and TB clinic, and ICU, ^***^= P-value < 0.001, ^**^= P-value < 0.01, ^*^= P- value < 0.05.

## Discussion

The magnitude of burnout among health professionals working in public health institutions in the Dire Dawa City administration was investigated in this study. Accordingly, the magnitude of burnout was 54.1%, which corresponds to the findings of a study in Iran (52.9%) (20), Ethiopia (50.4%) (21), and Senegal (55%) (26). This result, however, is also higher than that of previous studies conducted in Ethiopia: 36.7%, 34.0%, 47.6% (22, 27, 28), Egypt (24.9%) (29), and China (2.46%) (30). This discrepancy could be attributed to participants' socio-cultural and demographic factors as well as the country's healthcare system structure. It could also be related to the difference in the study settings. Other studies included only single institutions (20, 21, 27, 28) or -one profession (22, 26, 28–30), while this study included both hospitals and health centers as well as multi-professions.

In this study, 64.7% of the respondents had high emotional exhaustion. This finding is consistent with the findings of research done at Debra Berhan University hospital (61.8%) (22). However, it is higher than the studies carried out in Iran (55.3%) (20) and China (24.83%) (30). Among participants, 91.4% had high depersonalization, which is in line with the findings of a study done in Iran (90.5%) (20). However, this finding is higher than the results of studies undertaken at Debra Berhan University hospital (47.9%) (22) and China (6.21%) (30). Furthermore, according to the results of this study, 88.2% of the participants had low personal accomplishments. This result is lower than that of an Iranian study (98.9%) (20). However, it is higher than research conducted at Ethiopia's Debra Berhan University Hospital (59.7%) (22) and China's Hubei (33.99%) (30). This disparity might be due to the differences in methodology used to define the three components of burnout. It could also be due to differences in study areas and socio-cultural differences among study participants.

When comparing health professionals working in hospitals to those working in health centers, the risk of burnout was 3.55 times higher. This finding is congruent with a study conducted in Malawi (31). The reason for this could be that hospitals have a higher patient flow and a lower patient to health professional ratio than health centers, which can result in a high workload. That is one reason for increased health professionals' stress and exhaustion. In fact, an overburdened work schedule, or having too little time and resources to complete a task, is a key cause of burnout (22). When work is so quick, employees lose their sense of belonging and their performance degrades because of lack of regular sleeping or eating routines due to the enormous job load.

Those in the age groups of 30–39 years and ≥40 years had 1.9 times and 3.98 times higher risk of burnout than those in the age group of below 30 years. This finding is backed up by findings from other studies done in the rural parts of Ethiopia (6). When comparing health workers with 6–11 years of experience to those with 5 years or less, the risk of burnout was nearly two times higher. This finding is in line with the findings of a study undertaken in Ethiopia (21, 25). This could be explained by the fact that when people stay at the same job for a long time, there is nothing new to do, making the job monotonous. Furthermore, because the individual has worked in the same job for a long time, there is a risk that they will overwork themselves and become exhausted.

Female health workers were 2.41 times more likely to experience burnout than male health professionals to experience it. This result contradicts the findings of research done at Gondar University hospital (32), and Malawi (31). When compared to married health professionals, widowed health professionals had 3.39 times the risk of burnout. This finding is in line with the results of research conducted in North Showa, Amhara, Ethiopia (25). One possible explanation for this could be the socio-cultural aspects of being a woman and of marital status. Women in many African communities have additional responsibilities outside of their work, both socially and culturally. This might cause stress and weariness. Moreover, having an unstable marital status might lead to additional duties, which can lead to increased stress and a loss of interest in one's current employment.

Compared to nursing professionals, midwives, medical laboratories, public health officials, and other health professionals, they had a higher risk of burnout. Compared to working in an adult OPD, working in a pediatrics unit had a higher risk of burnout. Such differences could be explained due to the nature of their profession and related workload. Those who had the intention of leaving work had around 2.28 times higher odds of burnout compared with their counterparts. This finding is in line with the study conducted in the Amhara region (21). This could be explained by the fact that professionals who are planning to leave their jobs experience sentiments of dissatisfaction and frustration, which lead to in personal non-achievement and eventually changing careers.

Those who used at least one substance had 2.24 times the risk of burnout, and those who worked without supervision had 4.65 times the risk of burnout. This finding is supported by a study conducted in Malawi (31, 33–35). This could be due to the fact that substance use can lead to depression, tension, and reliance. Every profession necessitates the employee's commitment and time, and substance users may find it difficult to meet these needs. This could result in lower job performance and weariness. Moreover, job supervision is important for helping employees adapt to the working environment, so that it will reduce the challenges and stress in the working area and have a significant impact on the burnout of staff.

The odds of burnout were 5.69 and 2.74 times higher among health professionals who earn an average monthly payment of <10,000 ETB and between 10,000 and 15,000 ETB compared to those who earn above 15,000 ETB per month, respectively. A study conducted in Ethiopia backs up this finding (17). This could be explained by the fact that the income (incentive) of an employee is one of the motivational factors for conducting job effectively.

### Limitations of the study

There are certain limitations to this finding. Because it is a cross-sectional study, it can only reflect health professionals' experiences at the time of evaluation; hence, a causal relationship between health professional burnout and its predictors cannot be established. The other is that the study only included health professionals who worked in public health facilities. As a result, this may not be representative of health professionals that work in private health facilities.

## Conclusion

The findings revealed that the majority of health professionals working at Dire Dawa public health facilities had burnout. Working at the hospital, being 40 or more years of age, being female, being widowed, having an intention to leave work, using at least one substance, having 6–11 years of work experience, having no job supervision, earning an average monthly payment of <10,000 ETB and between 10,000 to 15,000 ETB, being a midwife, medical laboratory, and public health, and working in the pediatric unit were significantly associated predictors of burnout. We recommend the health facility manager focus on the female health professional's wellbeing to make them ready to give quality care. Establish mechanisms to improve staff retention; create different incentive mechanisms that increase staff income; and conduct regular supervision.

## Data availability statement

The original contributions presented in the study are included in the article/supplementary material, further inquiries can be directed to the corresponding author.

## Ethics statement

The studies involving human participants were reviewed and approved by the Institutional Health Research Ethical Review Committee (IHRERC) at Haramaya University. The patients/participants provided their written informed consent to participate in this study.

## Author contributions

All authors contributed to the study's conception and design, as well as the acquisition, analysis, interpretation of data, drafting and revising of the article, gave final approval of the version to be published, agreed to submit it to the current journal, and agreed to be accountable for all aspects of the work.

## Conflict of interest

The authors declare that the research was conducted in the absence of any commercial or financial relationships that could be construed as a potential conflict of interest.

## Publisher's note

All claims expressed in this article are solely those of the authors and do not necessarily represent those of their affiliated organizations, or those of the publisher, the editors and the reviewers. Any product that may be evaluated in this article, or claim that may be made by its manufacturer, is not guaranteed or endorsed by the publisher.

## Abbreviations

AOR: adjusted odds ratio
COR: crude odds ratio
CSA: central statistical agency
CI: confidence interval
DP: depersonalization
EDHS: Ethiopian Demographic and Health Survey
EE: emotional exhaustion
ETB: Ethiopian Birr
HSS: Human Service Survey
MBI: Maslach's Burnout Inventory
PA: personal accomplishment
SD: Standard deviation
SPSS: Statistical Package for the Social Sciences
WHO: World Health Organization.
